# Niche and fitness differences determine invasion success and impact in laboratory bacterial communities

**DOI:** 10.1038/s41396-018-0283-x

**Published:** 2018-09-25

**Authors:** Shao-peng Li, Jiaqi Tan, Xian Yang, Chao Ma, Lin Jiang

**Affiliations:** 1 School of Biological Sciences, Georgia Institute of Technology, Atlanta, GA 30332 USA; 2 Anhui Province Key Lab of Farmland Ecological Conservation and Pollution Prevention, School of Resources and Environment, Anhui Agricultural University, 230036 Hefei, Anhui China; 3 Present Address: Department of Biological Sciences, University of Pittsburgh, Pittsburgh, PA, 15260 USA

**Keywords:** Microbial ecology, Community ecology

## Abstract

There is increasing awareness of invasion in microbial communities worldwide, but the mechanisms behind microbial invasions remain poorly understood. Specifically, we know little about how the evolutionary and ecological differences between invaders and natives regulate invasion success and impact. Darwin’s naturalization hypothesis suggests that the phylogenetic distance between invaders and natives could be a useful predictor of invasion, and modern coexistence theory proposes that invader-native niche and fitness differences combine to determine invasion outcome. However, the relative importance of phylogenetic distance, niche difference and fitness difference for microbial invasions has rarely been examined. By using laboratory bacterial microcosms as model systems, we experimentally assessed the roles of these differences for the success of bacterial invaders and their impact on native bacterial community structure. We found that the phylogenetic distance between invaders and natives failed to explain invasion success and impact for two of three invaders at the phylogenetic scale considered. Further, we found that invasion success was better explained by invader-native niche differences than relative fitness differences for all three invaders, whereas invasion impact was better explained by invader-native relative fitness differences than niche differences. These findings highlight the utility of considering modern coexistence theory to gain a more mechanistic understanding of microbial invasions.

The last decade has seen a surge in the number of studies that documented worldwide invasion of microorganisms. The invasion of fungi [1, 2], algae [3], protists [4], and bacteria [5–7], which has been reported for various ecological systems (reviewed by [8]), is known to alter the structure and functioning of native communities [9]. Identifying the mechanisms of invasion in microbial communities thus has become an important objective of microbial community ecology [10, 11]. The majority of microbial invasion literature could be categorized into two classes: invader-centric research and resident community-centric research [10]. The invader-centric research focuses on identifying particular traits that characterize successful invaders (e.g., [12, 13]). By contrast, the resident community-centric research involves studying the properties of the resident (native) community that determine its susceptibility to invasion (e.g., [14, 15]). However, there is now an increasing recognition that integrating these two perspectives could offer new insights [16, 17], given that invasion outcomes may depend on the invader-native evolutionary and ecological similarities and differences [18].

The earliest idea linking invader-native differences to invasion outcome can be traced back to Charles Darwin. In his naturalization hypothesis, Darwin [19] predicted that invaders should be less successful in communities that contain their close relatives. The rationale behind the hypothesis is that the invaders and their closely related natives [i.e., low invader-native phylogenetic distance (PD)] tend to occupy similar niches, and thus compete strongly, reducing the chance of successful invasion (Fig. 1a). By the same logic, invaders are expected to impose stronger impacts on their more closely related natives due to their larger niche overlap [20] (Fig. 1a). An appreciable number of empirical studies have evaluated Darwin’s naturalization hypothesis, but results are often mixed, making it difficult to make general conclusions [21–23]. Moreover, most of these studies have focused on plant invasion (reviewed by [22, 24]), and significant gaps in our knowledge on the predictive ability of phylogeny for microbial invasions remain.

**Fig. 1:**
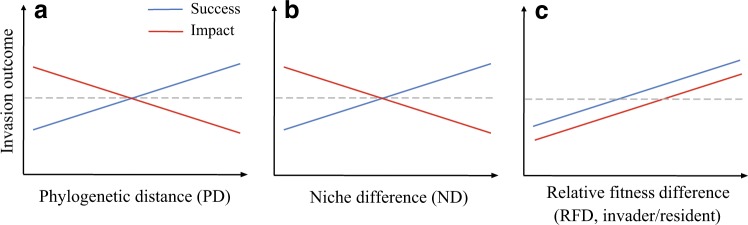
A conceptual diagram illustrating the influence of phylogenetic distance, niche difference, and relative fitness difference on invasion success and impact. Darwin’s naturalization hypothesis proposes that close relatives often occupy similar niches and compete strongly. Therefore, invaders should be less successful but produce stronger impacts in communities that contain their close relatives (**a**). Modern coexistence theory proposes that invader-native niche and fitness differences combine to determine invasion outcomes. Niche difference between invaders and natives reduces the strength of their competitive interactions. Therefore, increasing invader-native niche difference promotes the success of the invaders but hinders their impacts on natives (**b**). Fitness difference reflects competitive hierarchy that prevents species coexistence. Therefore, relative fitness difference, here measured as the fitness advantage of invaders over the natives, could enhance both invasion success and their impacts on native species (**c**). Together, exploring the relative importance of phylogenetic distance, niche and fitness differences on invasion success and impact would broaden our understanding of the mechanisms driving microbial invasions

Another framework that integrates species’ ecological differences into the study of invasion is modern coexistence theory, which suggests that invader-native niche and fitness differences combine to determine invasion outcomes [25, 26]. Species’ niche differences (ND), which cause species to limit themselves more than their competitors, would be predicted to stabilize the coexistence between invaders and natives. Relative fitness differences (RFD), which represent the differences in competitive ability among species, favor the competitive exclusion of species with lower fitness. According to this framework, increasing invader-native ND would increase the success of the invaders but decrease their impacts on native communities, due to the reduced strength of invader-native competitive interactions (Fig. 1b). On the other hand, both invasion success and impact would be expected to increase with the fitness advantage of the invaders over the natives (Fig. 1c). Together, invasion outcome would depend on the relative importance of invader-native ND and RFD. Notably, although the roles of ND and RFD in species coexistence have received much recent attention [27–30], the importance of these two aspects of ecological differences for invasion success and impact in microbial communities is poorly understood.

Darwin’s naturalization hypothesis and modern coexistence theory highlight the potential importance of invader-native evolutionary and ecological differences for biological invasions. Here, we used a simple laboratory experiment to explicitly evaluate the relative importance of these differences on invasion outcome (Fig. S1). The experiment subjected laboratory microcosms containing bacteria collected from a single source to the invasion of three non-indigenous bacterial species. Recent microbial invasion work has highlighted the utility of bacterial microcosms in developing a mechanistic understanding of invasion processes (e.g., [7, 16, 31]). The use of bacterial microcosms allowed us to quantify species ND and RFD with relative ease, as demonstrated by previous work with freshwater microalgae [30] and bacteria [32]. Building on previous work that applied modern coexistence theory to plants and algae [29, 30, 33, 34], we aimed to determine what aspects of invader-native evolutionary and ecological differences (i.e., PD, ND, and RFD) have stronger influences on bacterial invasion success and impact.

## Methods

### Bacteria and microcosms

Our experiment used a total of 11 bacterial species collected from freshwater ecosystems. We considered eight naturally co-occurring species, which were repeatedly isolated from a single pond—Lake Clara Meer in Piedmont Park of Atlanta, GA, USA, as natives. The frequent detection of these species from water samples suggests that they are not uncommon in the source lake, although the degree of their numerical dominance is unknown. These species exhibit distinct colony morphologies, allowing us to quantify their abundance via agar plate counts. The other three species (*Bacillus cereus*, *Serratia marcescens*, and *Staphylococcus pasteuri*), which are known to colonize a wide variety of habitats, served as invaders. We identified these species via sequence analysis of the 16S rRNA gene.

Microcosms were 25 mL capped test tubes, each filled with 6 mL of growth medium. The growth medium was made of four basic carbohydrates substrates—glucose, fructose, mannitol, and glycerol (2.50 g each dissolved in 1 L deionized water), along with tryptone (1.50 g/L), as the nitrogen source, 0.50 g K_2_HPO_4_, 0.50 g NaCl, and 0.30 g Mg_2_SO_4_. Using four basic carbohydrate substrates can effectively minimize habitat complexity, which allowed more accurate estimation of niche and fitness differences. We dissolved all the ingredients in deionized water, distributed the medium into each microcosm, and autoclaved the medium for 45 min before the experiment. During the experiment, all microcosms were incubated on a shaker at 220 rpm at room temperature (~22 °C).

### Phylogeny

**Fig. 2:**
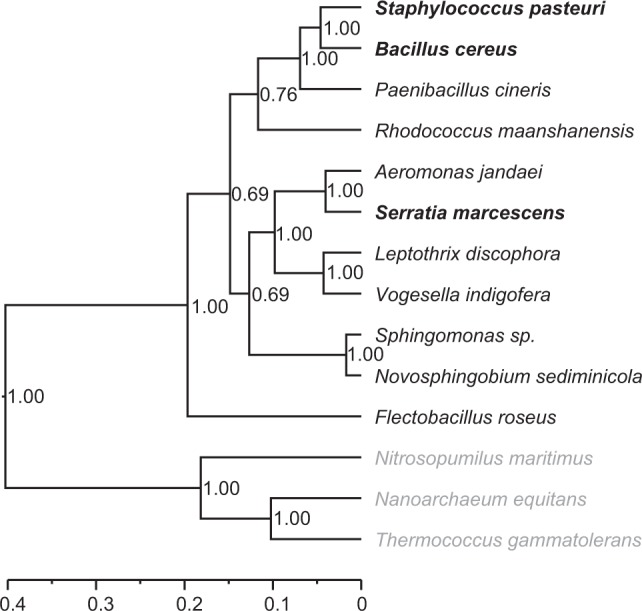
Bayesian phylogeny of the bacterial species used in this study. The phylogeny shows eight native bacterial species, three invaders (bold), and three out-group species (gray). The tree was constructed based on the 16S rRNA genes. The scale for branch length is shown below the phylogenetic tree. Scores on nodes indicate the posterior probability

We constructed phylogenies of the 11 bacterial species based on their 16S rRNA gene sequences. After sequencing the 16S rRNA, we aligned sequences using the program MUSCLE v3.8.31 [35], and selected the best-fit models of nucleotide substitution by the program jModeltest 2.1.10 [36]. We used the sequences of three archaeal species (i.e., *Nitrosopumilus maritimus*, *Nanoarchaeum equitans*, and *Thermococcus gammatolerans*) as the out-groups. We then constructed two ultrametric trees of these 14 species using the Bayesian and maximum likelihood approaches, following the method of Li et al. [20]. The Bayesian phylogeny was reconstructed using the program BEAST v1.8.4 [37]. The Bayesian MCMC chain was run for 10 million generations, and convergence was checked using Tracer version 1.6 (http://beast.bio.ed.ac.uk/Tracer). The consensus tree with the maximum clade credibility from the posterior distribution was used to quantify phylogenetic patterns using Tree Annotator 1.8.4 [37]. We also generated a maximum likelihood phylogeny using the program PHYML 3.0 with a BIONJ starting tree [38]. We then calculated the PD between invaders and natives by summing the length of the intervening branches between them. As the two phylogenies generated highly correlated PD values (Mantel test of pairwise PD: *r* = 0.998, *P* < 0.001), we only report results based on the Bayesian phylogeny (Fig. 2).

### Quantifying niche and RFD

The niches of microorganisms have often been assessed by the pattern of their use of provided carbon resources (e.g., Biolog; [7, 9]), which ignores other niche axes (e.g., spatial niches) and resources provided as metabolic products of other microbes (i.e., cross-feeding). Genomic data have also been used to infer species’ niches, providing information on the functions that can be potentially performed by microbes [39], but linking such information to ecological processes (e.g., invasion) has been difficult. Here we quantified invader-native ND and RFD through mutual invasion experiments (Fig. S1a), following previous work [30, 32, 40]. This approach measures ND based on species’ sensitivities in growth rate to competition (see below), and, therefore, represents the overall ND among species. The ND and RFD measures via this approach are also on the same scale, facilitating the comparison of their relative importance. For each invader-native pair, we first introduced 10 μL stock cultures (~10^4^ individuals) of the invaders to native-free microcosms. We also introduced the same number of invader into microcosms where the native species had established for 48 h, when the native had already achieved steady-state populations. Each treatment was replicated three times. The populations of the invader were sampled twice, at hour 6 and 14 after invasion, respectively, when all the populations were still in the exponential growth phase. Population densities at hour 6 and 14 were then used to calculate its per capita growth rate in the absence (μ_alone_) and presence (μ_invading_) of the native species. We averaged the growth rates of the three replicates to calculate the invader’ sensitivity (*S*_1_) to the native species, which was defined as: *S*_1_ = (μ_alone_ − μ_invading_) / μ_alone_. Likewise, we introduced 10 μL stock cultures of the natives (100 μL for *Flectobacillus roseus*, *Rhodococcus maanshanensis*, and *Leptothrix discophora*) to invader-free microcosms and the invader steady-state cultures, and calculated their growth rates and sensitivity (S_2_) to the invader. *F. roseus*, *R. maanshanensis*, and *L. discophora* had larger inoculum sizes because their stock culture population density (~10^5^ CFU/mL) was less than that of other native species (~10^6^ CFU/mL). By doing so, we controlled the initial cell densities of all bacterial species at the time of inoculation, ensuring that they were below 2% of their carry capacity. All species showed reduced growth rates in the presence of competitors than in monoculture (sensitivity > 0), indicating the presence of interspecific competition between all invader-native combinations. We calculated invader-native ND and RFD based on these sensitivities in short-term growth rate to competition (S_1_ and S_2_), by recognizing that large ND between invaders and natives would result in small values of S_1_ and S_2_, and large RFD would result in large differences in the values of S_1_ and S_2_ [40]. Following Carroll et al. [40] and Narwani et al. [30], ND was calculated as one minus the geometric mean of sensitivities:

1 $${\mathrm{ND}} = 1 - \sqrt {S_1S_2}$$

RFD was calculated as the geometric standard deviation of sensitivities:

2 $${\mathrm{RFD}} = \sqrt {S_2/S_1}$$

If RFD > 1, the fitness of the invader is greater than the native species, while RFD < 1 indicates the opposite.

### Experimental design

We assembled native bacterial communities that included all one-species monocultures and all possible two-species polycultures of the eight native bacterial species. We replicated each native community 15 times, for a total of 540 microcosms. At the beginning of the experiment (hour 0), we inoculated the eight native species into their designated microcosms, by transferring 10 μL (100 μL for *F. roseus*, *R. maanshanensis*, and *L. discophora*) of their stock cultures into the 25 mL tubes filled with 6 mL of growth medium. We allowed the native communities to equilibrate for 48 h before subjecting them to invasion. To determine native species composition before invasion, we destructively sampled 108 microcosms, with three replicates for each native community. Of the remaining 432 microcosms, 324 microcosms (36 treatment combinations × 3 different invaders × 3 replicates) were challenged with a single invader at hour 48, and the other 108 microcosms (36 treatment combinations × 3 replicates) were left as controls (Fig. S1b). The 10 μL stock cultures of the three invaders were inoculated into their designated microcosms in the same way as the natives. The experiment continued for another 48 h to allow for the establishment and growth of invader populations. Final sampling of the 432 microcosms was conducted at hour 96 to estimate the abundance of both native and invader species. Therefore, samples were collected twice, at hour 48 and 96 after the inoculation of resident species, respectively (Fig. S1). Throughout all the experiments, the population density of each bacterial species was estimated by plating serially diluted samples (five dilution levels from 10^2^ to 10^6^) onto agar plates and counting the number of colonies at appropriate dilution levels after three to six-day incubation.

### Data analysis

For the two-species native communities, the PD, niche and RFD of the invader to the recipient community were calculated as the mean phylogenetic distance (MPD), mean ND and RFD between the invader and the two native species. The results are qualitatively the same if we calculated the PD, ND, and RFD of each invader to its closest (or most abundant) native species in the recipient communities. Microbial invasions can be divided into sequential processes including introduction, establishment, growth and spread, and impact [11]. In our study, invasion success and impact were measured by the final performance of invaders and natives at hour 96. Invasion success was represented by the establishment (failure = 0, success = 1) and abundance of the invaders in the established microcosms. Abundance was measured by the natural logarithm transformed long-term population density (ln [x + 1]) of the invaders. Invasion impact was measured by the invasion-induced changes in the structure of native communities (i.e., changes in the abundance of native species due to invasion; [41, 42]), which was quantified as:

3 $$\sqrt {\left( {D_{i,{\mathrm{invaded}}} - D_{i,{\mathrm{control}}}} \right)^2 + \left( {D_{j,{\mathrm{invaded}}} - D_{j,{\mathrm{control}}}} \right)^2}$$

where *D*_*i*,invaded_ and *D*_*i*,control_ are the densities of native species *i* in invaded and control microcosms, and *D*_*j,*invaded_ and *D*_*j,*control_ are the densities of native species *j* in invaded and control microcosms. For communities that comprised one native species, this formula reduced to the absolute differences in the native species density between invaded and control microcosms.

For each invader, we first used ordinary least squares (OLS) regressions to assess the relationship among pairwise PD, ND, and RFD for the 24 invader-native combinations. We then used logistic regressions to assess the effects of MPD, ND, and RFD on invader establishment (failure = 0, success = 1). We further used OLS regressions to assess the effects of species richness (i.e., monocultures versus 2-species polycultures), MPD, ND, and RFD on invasion success (abundance of invaders) and impact (changes in native community structure).

In addition to regressions, we also preformed Bayesian phylogenetic mixed models, which account for phylogenetic non-independence as well as allow us to consider three invaders together, using the package MCMCglmm [43] in R [44]. To account for phylogenetic non-independence, we included species identity and phylogeny as random factors in the models. For ND and RFD, PD was considered as a fixed explanatory variable. For invasion success and impact, species richness, MPD, ND, and RFD were considered as fixed explanatory variables. In models with binary response variables (invader establishment), we followed the standard procedure and fixed residual variance to 1 [43]. In models with continuous response variables (ND, RFD, invader abundance, and impact), we specified a prior of an inverse-Wishart distribution for the random effects and residual variance components. We ran each model for five million iterations with a burn-in period of one million iterations and a thinning interval of 500. We used visual inspection of traces, as well as the Gelman–Rubin test, to assess model convergence. We considered all possible models containing different combinations of explanatory variables, and the best-fit models were identified based on deviance information criterion (DIC). As DIC may lead to models overfitting the data, we determined which single variable best explained invasion success and impact, and assessed the strength and significance of individual terms within the best models. All statistical analyses were performed in R version 3.3.2 [44].

## Results

### From PD to ND and RFD

**Fig. 3:**
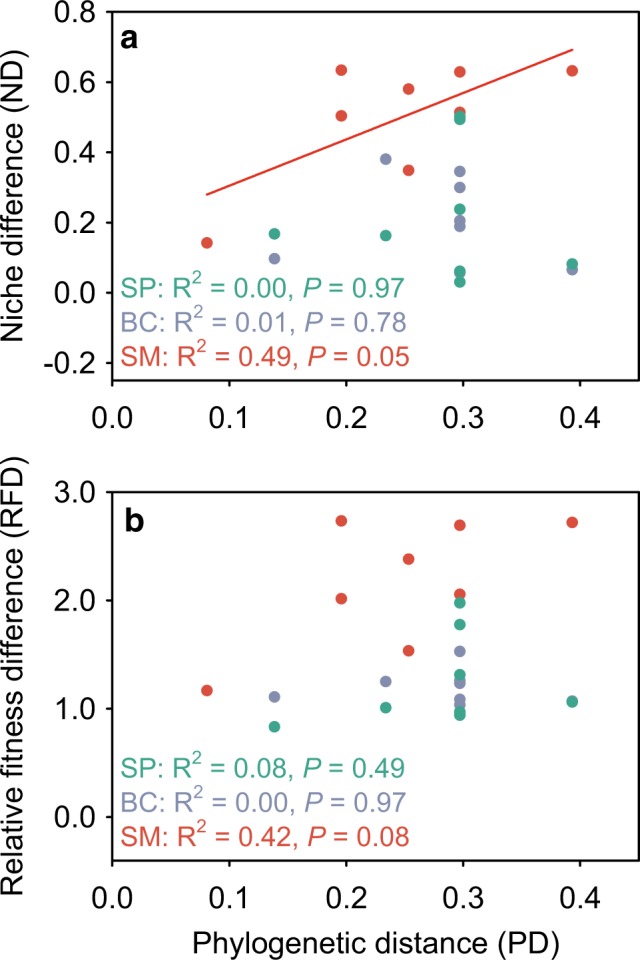
Invader-native niche differences (**a**) and relative fitness differences (**b**) in relation to phylogenetic distances. We quantified niche differences and relative fitness differences of all the 24 pairwise invader-native combinations (3 invaders × 8 natives). Different invaders are differently colored: *Staphylococcus pasteuri* (SP, green), *Bacillus cereus* (BC, purple), and *Serratia marcescens* (SM, red). Data are shown along with OLS regression lines if significant (*P* < 0.05)

For both *B. cereus* and *S. pasteuri*, ND and RFD were unrelated to PD (OLS regression: *df* = 6, R^2^ < 0.10, *P* > 0.40; Fig. 3). For *S. marcescens*, ND and RFD marginally increased with PD (OLS regression: *df* = 6, R^2^ = 0.49, *P* = 0.052 for ND; *df* = 6, R^2^ = 0.42, *P* = 0.082 for RFD; Fig. 3), indicating that *S. marcescens* exhibited larger ND and RFD with its more distantly related natives. When the three invaders were considered together, PD was a poor predictor of ND (MCMCglmm, *P*_MCMC_ = 0.466, *N* = 24) and RFD (MCMCglmm, *P*_MCMC_ = 0.231, *N* = 24).

### Phylogenetic patterns of invasion success and impact

**Fig. 4:**
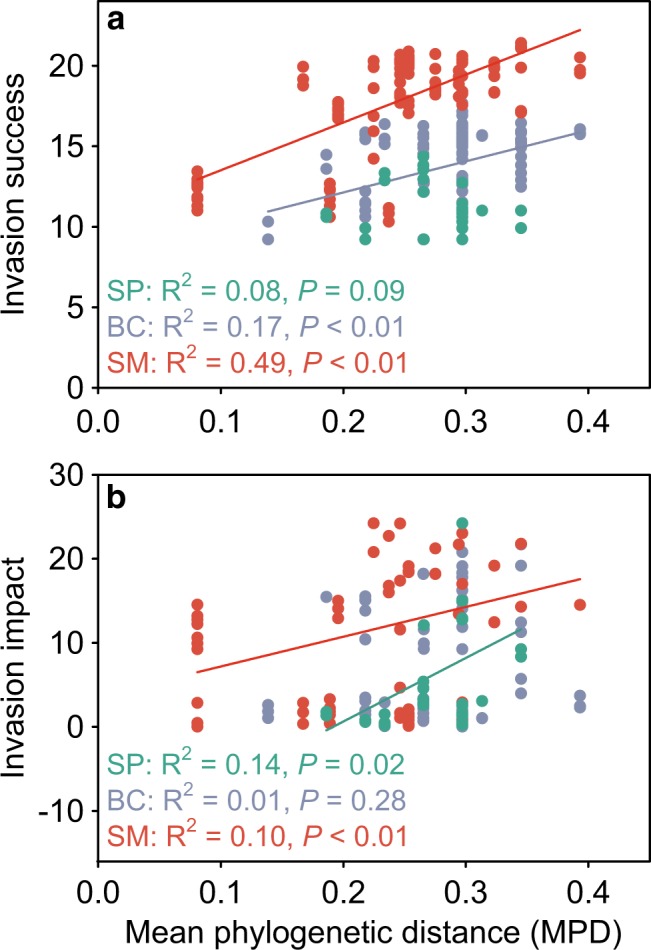
Invasion success and impact in relation to invader-native phylogenetic distances. Invasion success (**a**) is measured as the invader’s population density (ln-transformed), whereas invasion impact (**b**) is measured by the invasion-induced changes in the structure of native communities (see Methods). Different invaders are differently colored, and OLS regression lines are shown if significant

*S. pasteuri* and *B. cereus* established in 37 and 94 of the 108 invaded microcosms, respectively, while *S. marcescens* established in all invaded microcosms. The establishment probabilities of *S. pasteuri* and *B. cereus* were not related to MPD (logistic regressions, *df* = 106, *P* > 0.100; Fig. S2). The abundance of *S. pasteuri* showed a marginally significant decline with MPD, whereas the abundance of *B. cereus and S. marcescens* significantly increased with MPD (Fig. 4a). However, when the three invaders were considered together, MPD was unrelated to invaders’ establishment (MCMCglmm, *P*_MCMC_ = 0.254, *N* = 324) or abundance (MCMCglmm, *P*_MCMC_ = 0.710, *N* = 239).

The three invaders also differed in their relationship between MPD and invasion impact. For *B. cereus*, its impact on native community structures was not related to MPD (OLS regression: *df* = 92, R^2^ = 0.01, *P* = 0.281; Fig. 4b). For *S. pasteuri* and *S. marcescens*, their impacts increased with MPD (OLS regression: *df* = 35, R^2^ = 0.14, *P* = 0.020 for *S. pasteuri*; *df* = 106, R^2^ = 0.10, *P* < 0.001 for *S. marcescens*; Fig. 4b). When all three invaders were considered together, MPD slightly promoted the invasion impact on native community structure (MCMCglmm, *P*_MCMC_ = 0.025, *N* = 239).

### Relating ND and RFD to invasion success and impact

**Fig. 5:**
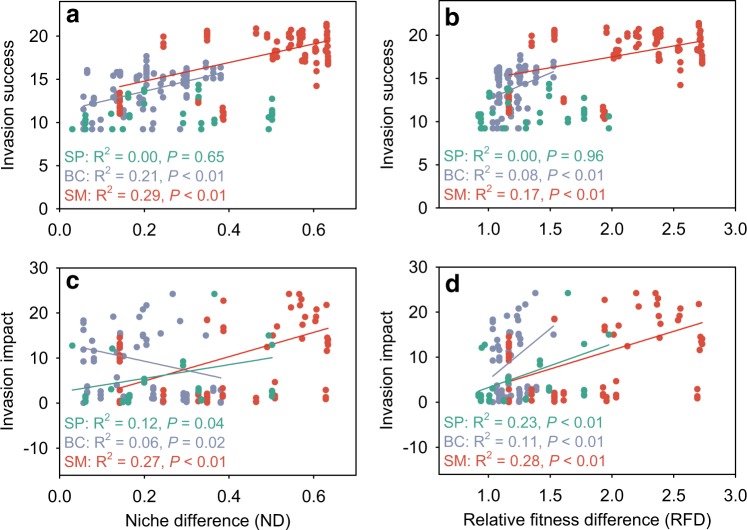
Invasion success and impact in relation to invader-native niche and relative fitness differences. The success (**a**, **b**) and impact (**c, d**) of the three invaders were regressed as functions of the mean invader-native niche and relative fitness differences in experimental microcosms. Different invaders are differently colored, and OLS regression lines are shown if significant

ND and RFD generally promoted invasion success (Fig. S3). The establishment probabilities of *S. pasteuri* and *B. cereus*, as well as the abundance of *B. cereus* and *S. marcescens*, significantly increased with invader-native mean ND, with one exception that the abundance of *S. pasteuri* was not related to ND (Fig. 5a, Fig. S2). Similarly, the establishment and abundance of invaders also increased with invader-native mean RFD, with one exception that the abundance of *S. pasteuri* was not related to RFD (Fig. 5b, Fig. S2). The relationship between ND and invasion impact, however, varied among the invaders. The impact of *S. pasteuri* and *S. marcescens* on natives significantly increased with ND, whereas the impact of *B. cereus* on natives significantly decreased with ND (OLS regression: *P* < 0.05 for all; Fig. 5c). In contrast, the invasion impacts on natives significantly increased with invader-native mean RFD for all three invaders (OLS regression: *P* < 0.01 for all; Fig. 5d), indicating that invaders had stronger impacts when they showed greater fitness than natives. For all three invaders, ND was a better predictor of invasion success than RFD, whereas RFD was a better predictor of invasion impact than ND (Fig. 5).

Model selection revealed that species richness (i.e., monocultures versus 2-species polycultures) and MPD were poor predictors of invasion success and impact, as they were excluded or among the least important variables retained in the best multivariate models (Table 1). Invasion establishment could be best predicted by ND in either multivariate or univariate models (Tables 1 and 2). The best overall regression model of invader abundance retained MPD, ND, and RFD as explanatory variables (Table 1), and ND was the single best predictor in the univariate model (Table 2). Both ND and RFD were significant in the best-fit multivariate model of invasion impact (Table 1), and RFD was the single best predictor of invasion impact (Table 2).

**Table 1:** The best-fit models explaining variation in invasion success and impact

Formula	Variable	Posterior mean	Low 95% CI	Upper 95% CI	*P*_MCMC_	DIC
Establishment (0,1)						
~ND						193.96
	**ND**	**9.42**	**5.87**	**12.89**	**<0.001**	
Success (ln-transformed density)						
~MPD + ND + RFD						1837.98
	MPD	9.83	−1.78	21.6	0.084	
	**ND**	**27.5**	**20.08**	**34.9**	**<0.001**	
	**RFD**	**−6.01**	**−9.12**	**−3.03**	**<0.001**	
Impact (change in the native community structure)						
~SR + ND + RFD						1599.86
	SR	1.87	−0.09	3.97	0.067	
	**ND**	**−34.1**	**−47.82**	**−19.37**	**<0.001**	
	**RFD**	**19.94**	**14.92**	**25.13**	**<0.001**	

Our list of candidate multivariate and univariate models included all possible combinations of the explanatory variables. Invasion success was represented by the establishment and abundance of the invaders in the invaded microcosms, while invasion impact was measured by the invasion-induced changes in the structure of native communities (see Methods). The invader identity and phylogeny were included in the MCMCglmm models as random factors, and the best models were ranked and selected by DIC. The significant variables in the best-fit models are highlighted in bold

*SR* species richness, *MPD* mean phylogenetic distance, *ND* niche differences, *RFD* relative fitness differences, *DIC* deviance information criterion

**Table 2:** Univariate models explaining variation in invasion success and impact

Variable	Posterior mean	Low 95% CI	Upper 95% CI	*P*_MCMC_	DIC	ΔDIC
Establishment (0,1)						
SR	−0.36	−1.29	0.55	0.450	225.30	31.34
MPD	−5.06	−24.63	17.80	0.254	223.63	29.67
**ND**	**9.42**	**5.87**	**12.89**	**<0.001**	**193.96**	**0.00**
RFD	4.54	2.74	6.46	<0.001	199.13	5.17
Success (ln-transformed density)						
SR	−0.50	−1.74	0.71	0.435	1920.40	82.42
MPD	6.55	−56.67	72.08	0.710	1896.19	58.21
**ND**	**15.27**	**11.87**	**18.63**	**<0.001**	**1849.68**	**11.71**
RFD	4.24	2.92	5.68	<0.001	1886.29	48.31
Impact (change in the native community structure)						
SR	1.15	−1.24	3.52	0.349	1672.89	73.03
MPD	29.76	6.53	50.00	0.025	1658.23	58.37
ND	15.47	9.03	21.93	<0.001	1655.14	55.28
**RFD**	**8.67**	**6.33**	**11.16**	**<0.001**	**1622.13**	**22.27**

The best univariate models were selected by the DIC, and compared to the best multivariate models in Table 1 using ΔDIC. These best models are highlighted in bold

*DIC* deviance information criterion

## Discussion

Understanding the mechanisms regulating microbial invasions represents an emerging challenge in microbial ecology. Within this context, species’ evolutionary and ecological differences have received much recent attention. For example, genotypic dissimilarity and functional dissimilarity of the resident communities have been shown to better predict bacterial invasions than resident genotypic and taxonomic richness [31, 45]. However, previous studies have largely focused on the evolutionary and ecological differences among natives, and how invader-native differences influence invasion outcome remains largely unexplored. On the other hand, modern coexistence theory has recognized the importance of ND and RFD for regulating species coexistence, and recent empirical studies supported the utility of this framework for predicting competitive outcomes in plant and algal communities (e.g., [28–30]). By extending this framework to bacterial invasions, we evaluated the relative roles of invader-native PD, ND, and RFD on invasion success and impact. We found that invader-native PD was a poor predictor of invasion success and impact for two of three invaders, whereas for all three invaders, invasion success was better explained by invader-native ND than RFD, and invasion impact was better explained by invader-native RFD than ND. Together, these results suggest that considering different aspects of invader-native differences would broaden our understanding of invasion mechanisms in microbial communities.

A central assumption of Darwin’s naturalization hypothesis is that closely related species occupy similar niches, and, therefore, invader-native PD could capture their ND and competitive intensity. Although empirical evidence for this assumption has been frequently reported, the generality of this assumption is still under debate [46]. We found inconsistent PD-ND relationships for the three invaders (Fig. 3a), which echoes the mixed patterns reported by the few studies that have quantified this relationship [29, 30, 32]. For example, phylogeny was a significant predictor of ND between the bacterium *Pseudomonas fluorescens* SBW25 and its bacterial competitors [32], but it failed to explain ND among freshwater green algae [30], as well as among California annual plant species [29]. These results indicate that the relationships between phylogeny and niches are complex, potentially depending on the tempo and mode of trait evolution, the phylogenetic scales considered, and the biogeographic history [47–49]. For example, we might expect phylogeny to be less informative of ND when evolutionary rates are rapid [48], and the phylogenetic niche conservatism may decline with clade age [50]. Indeed, microbial traits relevant to niche overlap and competition, such as carbon substrate utilization and organic phosphorus uptake, are generally shallowly conserved [49, 51]. Our study focused on a relatively broad phylogenetic scale (i.e., the 11 bacterial species are from four phyla), which may potentially explain why we did not find significant PD-ND relationships. Moreover, biogeographic history could also influence PD-ND relationships, such that species lacking coevolutionary histories could exhibit weaker PD-ND relationships than coevolved species [47]. Together, these complexities argue against the indiscriminate use of phylogeny to infer species’ niche similarity in resolving Darwin’ naturalization hypothesis. In our experiment, invader-native PD adequately captured ND and RFD for only one of the three invaders (i.e., *S. marcescens*), and we found strong effects of PD on both the success and impact of *S. marcescens* but not the other two invaders (Figs. 3 and 4). Therefore, the validity of Darwin’ naturalization hypothesis appears to depend on how invader-native PD translates into ND and RFD that combine to regulate invasion success and impact.

The theory of limiting similarity suggests that the high degree of invader-native niche overlap would hamper invasion success [52]. Most invasion studies have tested this theory by examining the similarity in functional traits between invaders and natives [53, 54], or their overlap in resource use [17, 45]. However, linking species trait differences to ND is challenging, given that species occupying distinct trait space can still occupy similar niches [55]. Characterizing resource consumption patterns is a more straightforward approach to quantifying niches of microorganisms. However, this approach focuses only on resource partitioning, and generally ignored other aspects of species’ niches (e.g., spatial, temporal, and trophic niches). Recent advances in modern coexistence theory provided us an alternative method that allowed the quantification of species niche and fitness differences through short-term mutual invasion experiments [30, 40]. By doing so, we found that the invaders established better and attained larger population sizes when they showed larger ND from the natives (Fig. 5a; Fig. S2), providing direct experimental support for the limiting similarity theory. Note that although modern coexistence theory suggests that both invader-native niche and fitness difference could be important for regulating invasion success [25], the importance of these two mechanisms has only begun to be evaluated (e.g., [27–30]). Our study provided direct evidence that ND better explained invader establishment and abundance than fitness differences (Tables 1 and 2), suggesting that invaders were more limited by their niche overlap with the natives than their own fitness when introduced into new habitats. This result is in line with recent empirical work reporting the more important role of ND, relative to fitness differences, for species coexistence in plant communities [27, 28]. The paucity of studies, however, calls for the need for future studies to test the generality of these results.

A predictive understanding of invasion impact on native communities is another important goal of invasion ecology. Recent studies have demonstrated that microbial invasion could produce strong impacts on the recipient communities. For example, the invasion of the fungal pathogen *Rhizoctonia solani* altered microbiome composition and stress-related gene expressions of the invaded rhizobacterial communities [2]. The invasion of *Escherichia coli* in soil microcosms, although often unsuccessful, changed the diversity, composition, and niche structure of the invaded soil microbial communities [9]. However, it remains a challenge to predict the magnitude of invaders’ impacts on different invaded communities. Recent studies from plant communities reported that the invaders imposed stronger impacts on their more closely related natives [20], or on the natives with larger niche overlap to the invaders (e.g., niche replacement hypothesis; [42]). However, the relative importance of different invader-native difference measures has not been compared. By simultaneously considering invader-native PD, ND, and RFD, we found that RFD better explained invasion impacts than PD and ND (Tables 1 and 2). Therefore, the fitness hierarchy of invaders to natives was more important than their niche overlap and phylogenetic relatedness in determining invasion impact in our experiment. This result is consistent with the findings on plant communities that the difference between native and invasive fitness (e.g., the differences in cover and height) was the most important determinant of invasion impact [56]. Note that in simple regressions, ND was positively associated with the impact of *S. pasteuri* and *S. marcescens* on natives (Fig. 5c), whereas in multiple regressions, which accounted for the effects of RFD, ND was generally negatively associated with invasion impact (Table 1). This result reflects the fact that RFD played a more important role in determining invasion impact than ND, such that invaders with greater fitness would produce stronger impacts on native communities, even if they had lower niche overlap. This result also supports the idea that simply quantifying the effect of ND on invasion success and impact without considering RFD, or vice versa, may yield inaccurate conclusions [57]. Together, these findings indicate that communities composed of species with relative lower fitness to invaders are likely more influenced by invasions, regardless of the invader-native niche overlap.

Despite being effective in demonstrating the relative roles of invader-native PD, ND, and RFD in bacterial invasions, several limitations of our study should be noted. First, our experiment, similar to many other studies of bacterial invasion (e.g., [7, 9, 14, 16, 17, 31]), considered any species that are not currently present in the resident community as potential invaders [10]. Therefore, the origins and the natural ranges of these invaders have not been fully considered. As evidence is accumulating that bacterial species do, in many cases, exhibit biogeographical patterns and dispersal limitation [58–60], further studies are necessary to examine how our results can apply to the invasion of microbes dispersing beyond their natural boundaries. Second, our experiment had no more than two species in native communities. We used this design in part because more diverse communities have greater chance containing both similar and dissimilar natives to the invaders, where different aspects of evolutionary and ecological differences (i.e., invader-native PD, ND, and RFD) often confound with species richness, making it difficult to distinguish their contributions to invasion success and impact. Future studies, however, should assess the robustness of our results using more diverse assemblages of invaders and natives. Third, in our experiment ND and RFD were measured based on short-term species growth rate data (specifically sensitivity in short-term growth rate to competition), following Narwani et al. [30], whereas invasion success and impact were measured based on long-term population abundances. One could argue, however, that species growth rate and abundance may not be independent from each other. Nevertheless, we found no relationships between species’ growth rate and steady-state abundance for the 11 bacterial species in monocultures (linear regression, df = 9, R^2^ = 0.24, *P* = 0.128). More broadly, bacterial growth rate is generally a poor predictor of their abundance in nature [61–64], probably because the two are differentially influenced by the same ecological factors (e.g., predation and viral lysis, [62]; bacterial niche breadth, [63]). It would be desirable to be able to directly quantify ND based on the dimensions of niches utilized, but this ideal approach is difficult to implement in many situations where the dimension of niches (e.g., spatial niches and cross feedings) is difficult to quantify or even unknown. Finally, the use of laboratory bacterial communities allowed us to quantify invader-native ND and RFD and compare their strength on invasion success and impact, illustrating the utility of species coexistence theory for predicting biological invasions in simple microbial communities. However, the estimation of ND and RFD remains difficult in more complex natural microbial communities. It remains to be seen whether and how differences in specific traits or trait combinations between natives and invaders could effectively translate into ND and RFD, which may provide a possible pathway for the predictive understanding of microbial invasions.

Our study shows that different aspects of invader-native evolutionary and ecological differences can differentially affect invasion outcome. At odds with Darwin’s naturalization hypothesis, we find that phylogeny is not a reliable predictor of invasion success and impact at the phylogenetic scale considered. However, by evaluating the roles of niche and fitness differences on invasion success and impact, our study reveals that these two aspects of invader-native differences are more important determinants of invasion success and impact, respectively. These findings illustrate the utility of applying modern coexistence theory for a more mechanistic understanding of microbial invasions.

## Electronic supplementary material

Supporting Information
