# Supplementary material for: Niche and fitness differences determine invasion success and impact in laboratory bacterial communities
Source: ISME J. 2018 Sep 25;13(2):402–12. doi: 10.1038/s41396-018-0283-x (PMC6331569; doi:10.1038/s41396-018-0283-x)
Supplement: Supplementary file 1 — Supporting Information [file 41396_2018_283_MOESM1_ESM.pdf]

Supporting Information for

Niche and fitness differences determine invasion success and impact in laboratory bacterial communities

Shao-peng Li, Jiaqi Tan, Xian Yang, Chao Ma & Lin Jiang

Supporting Information includes three supplementary figures.

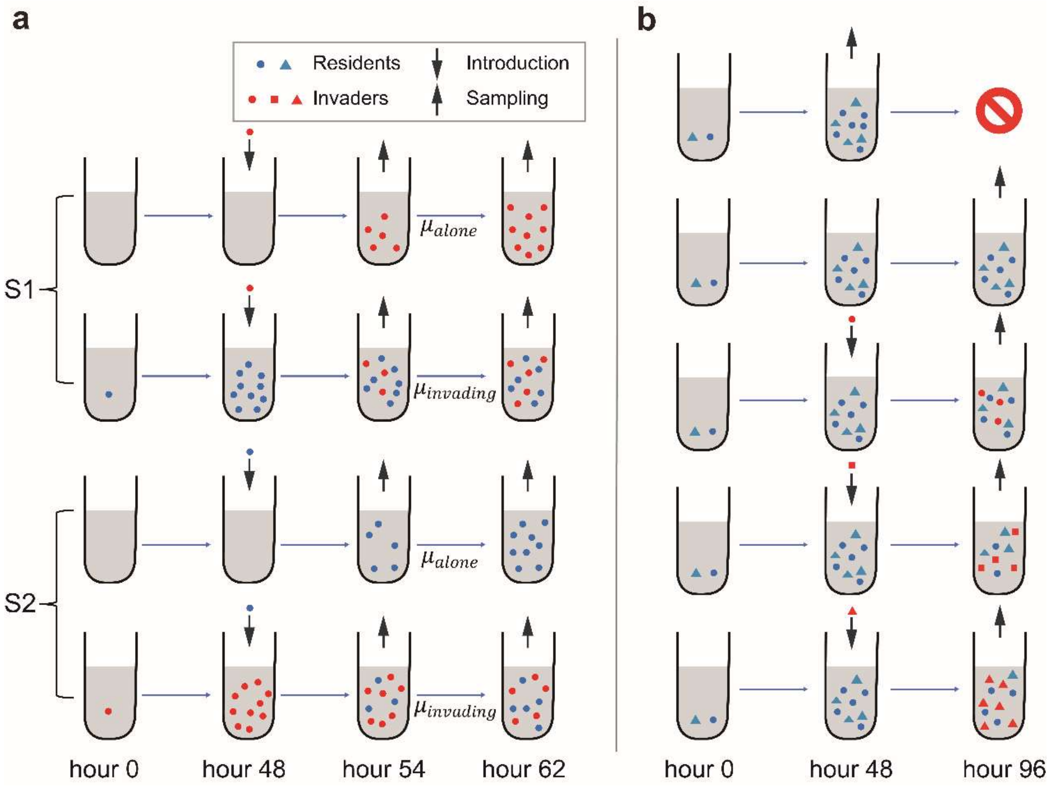

Figure S1. The conceptual diagram of the experimental design for (**a**) quantifying niche and relative fitness differences, and (**b**) testing the dependence of invasion success and impact on mean phylogenetic distance (MPD), niche differences (ND) and relative fitness differences (RFD).

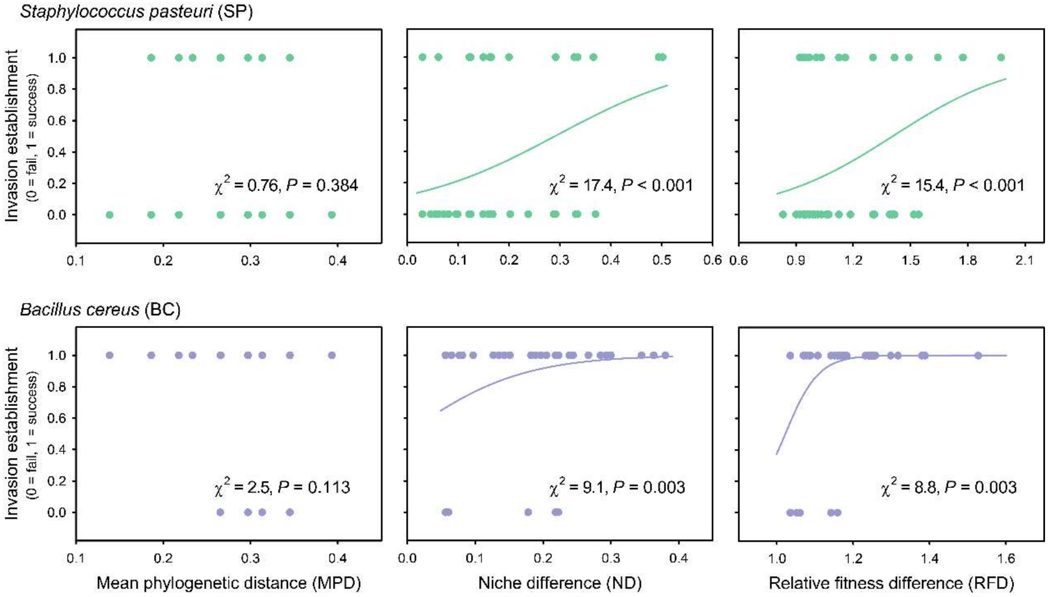

Figure S2. The dependence of invasion establishment on mean phylogenetic distance (MPD), niche differences (ND) and relative fitness differences (RFD). Invasion establishment is viewed successful if the invader attained above-zero density in the recipient communities. Different invaders are differently colored: *Staphylococcus pasteuri* (SP, green) and *Bacillus cereus* (BC, purple); *Serratia marcescens* (SM) is not shown as it established in all recipient communities. Statistical results are shown along with logistic curves if significant.

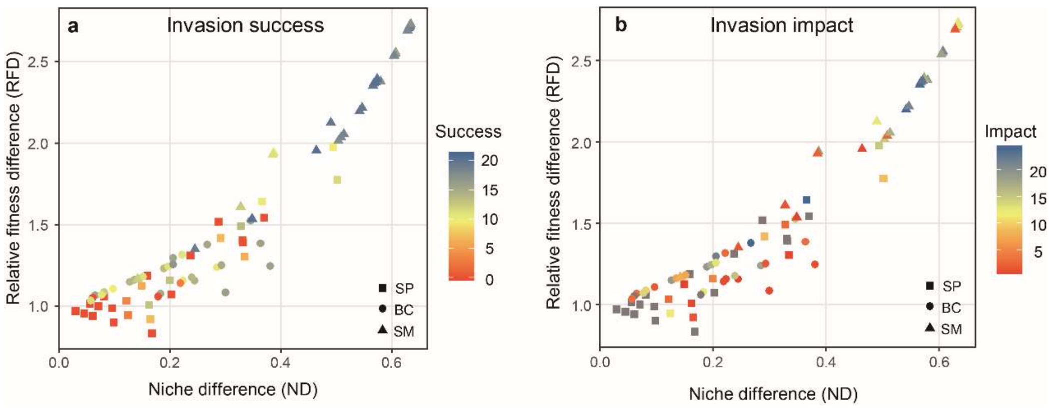

Figure S3. The relationship between invader-native niche differences and relative fitness differences, and their effects on invasion success (a) and impacts (b). The different symbols indicate different invaders. The color-coding of the dots indicates the level of success or impact. For invasion impact, the unsuccessfully invaded microcosms are colored in grey.
